# Plasma concentration of metformin and dexamethasone after administration through Osseogate

**DOI:** 10.1080/10717544.2016.1261380

**Published:** 2017-02-06

**Authors:** Hong-Kyun Kim, Young-Seok Park

**Affiliations:** Department of Oral Anatomy, Dental Research Institute and School of Dentistry, Seoul National University Seoul, Seoul, Republic of Korea

**Keywords:** Osseogate, implant-mediated drug delivery system, solubility, dexamethasone, metformin

## Abstract

*Background*: Osseogate is a novel drug delivery route through bone marrow involving a modified implant used as the drug delivery system. The purpose of this study was to evaluate the effects of individual drug physicochemical characteristics on the pharmacokinetic behavior when using the Osseogate as administration route.

*Methods*: Implant-mediated drug delivery systems (IMDDS) were installed in a total of 18 rabbits. After complete healing, water-soluble metformin hydrochloride was administered to one group (*n* = 9) while poorly soluble dexamethasone was administered to the other group (*n* = 9). The release patterns of each group were monitored for two weeks by measuring the plasma concentration of each drug.

*Results*: Both groups showed relative sustained release. However, metformin hydrochloride showed more rapid diffusion and early termination of drug release compared with dexamethasone.

*Conclusions*: The physicochemical properties of drugs significantly affected the release pattern through Osseogate. Further research is required to achieve controlled delivery using this route.

## Background

Since the development of novel drug molecules is not only time consuming but also substantially expensive, improving the safety to efficacy ratio of existing drugs is an attractive goal that has been investigated in a number of studies (Tiwari et al., 2012). These efforts include simple dose titration, slower delivery, controlled release, targeted delivery and individualized drug therapy, all of which can be categorized as improvement of either the drug itself or its carrier (Renugalakshmi et al., 2011). In this respect, a drug delivery system (DDS) can be defined as a formulation or a device that introduces a therapeutic substance into the body and maximizes its efficacy and safety by controlling every aspect of the drug’s pharmacokinetics and pharmacodynamics (Jain, 2008).

Drugs can be administered to the body through various anatomical routes, and important considerations should be given to the route of administration when devising a DDS. Traditionally, the oral route is the most prevalent for both conventional and novel drug delivery. Although there are many limitations of the oral route, the major advantage, namely the ease of administration, overwhelms many of its disadvantages. The second most prevalent route of administration is the parenteral route, which usually entails various types of injections. Aside from the advantage of rapid onset, many important drugs are only available in parenteral form. However, because it is an invasive form of delivery, the greatest drawback of the parental route is the accompanying pain (Sanders, 1990).

The selection of the administration route depends on the disease, the desired effect and available products. Specifically, it can be changed according to the intended range of effect, either for the whole system or just one target organ (Jain, 2008). The field of dentistry has a profound interest in drug delivery, typically aiming for regional effects, especially with respect to caries control (De Sousa et al., 2014), periodontal infections (Yao et al., 2014), local anesthesia (Silva de Melo et al., 2014) and delivery of bone morphogenic protein (Ramazanoglu et al., 2013).

The recently introduced implant-mediated drug delivery system (IMDDS) is somewhat different from the dentistry DDS applications described above, in that it is not limited to oral and maxillofacial purposes, but its main target is to achieve systemic delivery of drugs for the treatment of chronic disease (Park et al., 2014a,b). Specifically, IMDDS involves delivery of desired pharmacologic substances into the body via bone, a route referred to as Osseogate. This method has a number of promising features as a novel drug delivery route, the most notable of which is its ability to act as a permanent gateway to provide repeatable and sustainable drug release without the need for multiple needle injections (Park et al., 2016).

Several candidate drugs are being tested using Osseogate delivery and have thus far displayed satisfactory results (Park et al., 2014b). However, a great deal remains to be learned about the attributes of the Osseogate, the novel route of administration. Although the results of previous studies demonstrated sustained plasma concentrations of a target drug for up to eight weeks (Park et al., 2014a), it is possible that this was due to the unique properties of the drug being evaluated. Therefore, there is a need for continued evaluation of the Osseogate delivery patterns of several drugs with different physicochemical characteristics.

Metformin hydrochloride and dexamethasone are well-known drugs that are used on a long-term basis and are quite familiar among dentists. In addition, these two drugs exhibit significant differences, especially with respect to their solubility. In the present study, we compared the plasma concentration of metformin hydrochloride and dexamethasone after administration through the Osseogate route. Thus, the aim of this study was to evaluate the effect of drug physicochemical characteristics on pharmacokinetics using Osseogate delivery.

## Methods

### Animals

A total of 18, 10-week-old male New Zealand White rabbits, each weighing 2.5–3.0 kg, were used in this study. Animal selection, management and surgical protocols were conducted according to routine procedures approved by the Institutional Animal Care and Use Committee of Seoul National University, Seoul, Korea. Before the start of experiments, rabbits were quarantined and acclimated for seven days. Afterwards, rabbits were housed in separate cages and provided a standard rabbit diet and water *ad libitum*. Temperature and relative humidity were kept at 22 ± 4 °C and 50–60%, respectively, with a 12-h light/dark cycle.

### Implant-mediated drug delivery system

**Figure 1.**
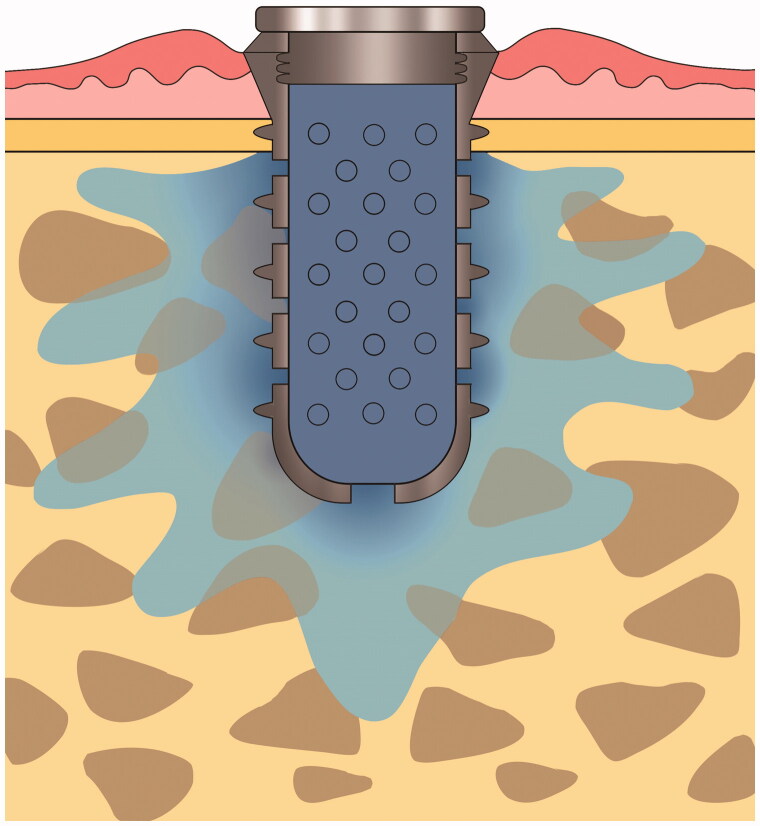
Implant mediated drug delivery system, which release drug through novel route of administration, Osseogate.

IMDDS devices were custom made by a milling machine using grade IV titanium to facilitate osseointegration. The fixture part of the IMDDS (diameter of 4.1 mm and length of 7.0 mm) consisted of external threads to facilitate initial stability in bone, while the remainder of the device consisted of a hollow cylindrical transmucosal implant with multiple diffusion holes. The drug cartridge, which consisted of either metformin or dexamethasone, was inserted inside the hollow cylinder. Multiple holes were located in both the circumferential axial wall and the apex of the fixture to allow for drug dispersion into the bone. The top of the fixture was equipped with a cover to separate the inside of the IMDDS from the external environment (Figure 1).

### Surgical procedures

Prior to bring rabbits to the surgical room, the hind legs were shaved. The animals were then anesthetized with an intramuscular injection of 10 mg/kg zolazepam and 0.15 ml/kg xylazine hydrochloride, and local anesthesia comprising 2% lidocaine with 1:100 000 epinephrine (0.5–1 ml/site) was applied around the surgical site. The site was accessed with 3-cm-long periosteal incision through the skin. The flat lateral surface of the proximal tibia metaphysis was selected for IMDDS installation. The IMDDS was placed after sequential site preparation using a 2.0-, 3.0-diameter twist drill and screw tap. Each rabbit received one IMDDS randomly in the left or right tibia. The surgical site was closed in layers with appropriate absorbable sutures. The surgery procedure was performed using aseptic technique. After surgery, each rabbit received a non-narcotic, non-steroidal analgesic agent for pain control and a broad-spectrum antibiotic for infection control.

### Loading of metformin hydrochloride and dexamethasone

Metformin and dexamethasone have relatively well-established pharmacokinetic properties. Metformin is a first line anti-hyperglycemic agent for type II diabetes mellitus patients, whereas dexamethasone is an efficient anti-inflammatory drug used in the treatment of several chronic diseases. Both metformin hydrochloride (PHR1084-500MG, Sigma-Aldrich, St. Louis, MO) and dexamethasone powder (D1756-1G, Sigma-Aldrich, St. Louis, MO) were prepared as 10 mg cartridges. A single cartridge of respective drug was then inserted into the implant and sealed with a cover screw (Figure 1).

### Measurement of the metformin and dexamethasone plasma concentration

Pharmacokinetic studies conducted by measuring drug plasma concentration are essential for understanding DDSs. In the present study, 3-ml blood samples were taken from the marginal vein of the ear at predetermined time intervals immediately after drug administration through a follow-up time of two weeks. Blood samples were collected in heparin tubes and divided into two 1.5 ml tubes for centrifugation. Plasma was taken for analysis after separation via centrifugation (3000 rpm, 10 min in 4 °C). The plasma concentration of each drug was determined using liquid chromatography tandem mass spectroscopy (LC–MS/MS System, AB SCIEX, Framingham, MA) at each time point. The study design was a complete data design in which each subject is sampled at all predefined time points.

### Statistical analysis

Serial measurements were correlated for each rabbit because of the repeated observations at each time point. To control for this correlated data structure, a mixed-effects analysis was applied. Statistical analyses were performed using the R statistical language, and *p* values < 0.05 were considered statistically significant, and were also assessed at the 0.01 level of confidence.

## Results

There were no abnormalities, mobility issues or inflammation of the implant sites during the observation period after placement.

**Figure 2.**
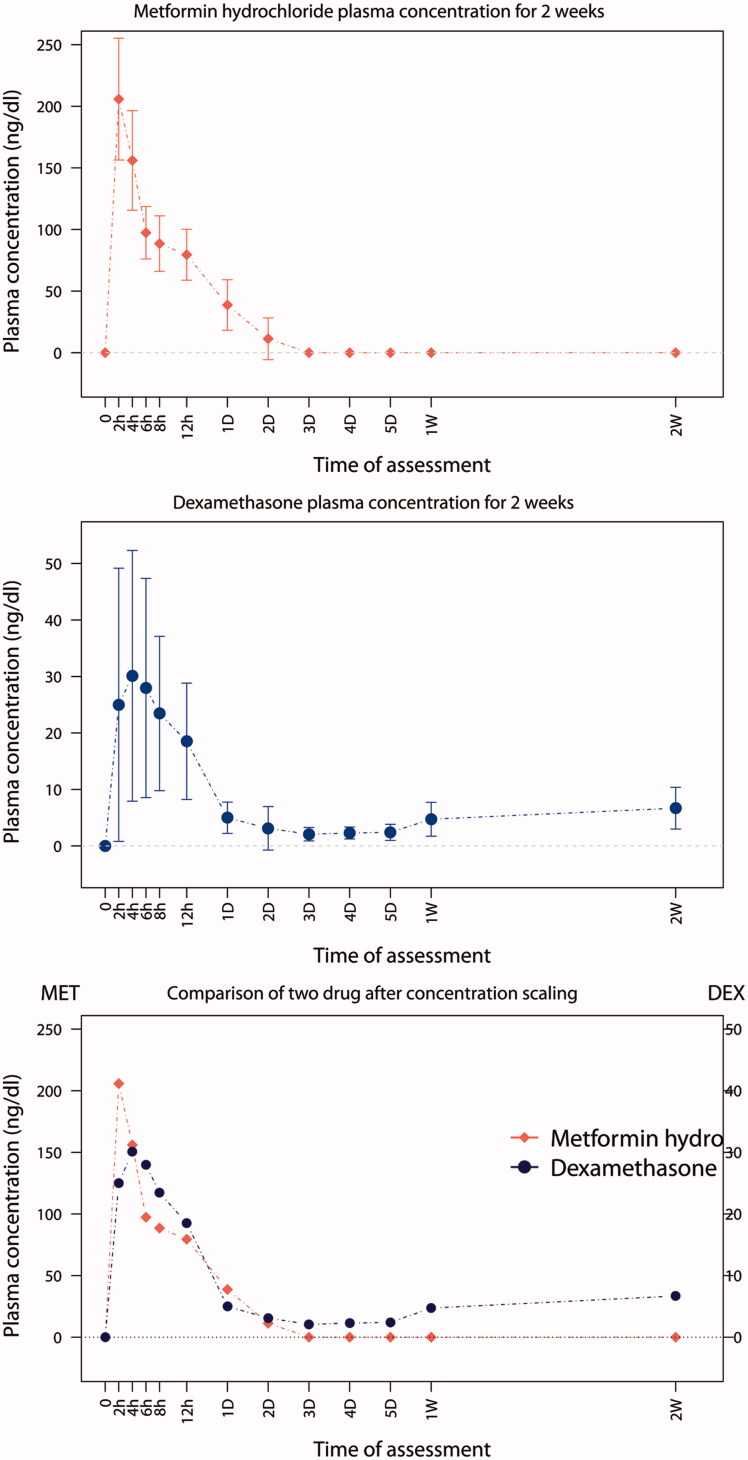
Plasma concentration of metformin hydrochloride and dexamethasone administered through Osseogate. The bottom is the superimposition of the two graphs after scaling the concentration.

The cumulative release profiles of each drug were obtained for a total of 18 rabbits. Considerable variation was observed in the plasma concentrations in each experimental rabbit. In the profiles of both drugs, two common features were readily apparent. First, the release profile demonstrated no lag time immediately after the drug administration. Second, there was a relatively sustained release pattern. In addition, we observed significantly different patterns between the two drugs over the observation period. Specifically, metformin hydrochloride reached a maximum plasma concentration at the first time measurement obtained two hours after administration, but was not detected in samples gathered at 72 h. In contrast, the peak plasma levels of dexamethasone occurred four hours after administration. The concentration was continuously sustained until the end of the observation period and showed even slight increasing tendency at the final measurements. Thus, some of pharmacokinetic parameters could not be obtained. Consistent with these data, the peak concentration of metformin hydrochloride was much higher than that of dexamethasone (Figure 2).

The area under the curve (AUC) is a reliable index for estimating drug bioavailability (Jain, 2008). The total AUC for two weeks of observation for metformin hydrochloride and dexamethasone and were 2.87 mg/ml·h and 1.87 mg/ml·h, respectively. Thus, the bioavailability of both drugs delivered through the Osseogate route was evident based on AUC data (Table 1).

**Table 1.** Mean pharmacokinetic parameters for metformin hydrochloride and dexamethasone administered via Osseogate.

	Metformin hydrochloride	Dexamethasone
*C*_max_ (ng/ml)	205.77	62.116
*T*_max_ (h)	2	4
AUC_last_ (ng/ml·h)	2874.02	1870.21
AUC_∞_ (ng/ml·h)	2874.02	NA^a^
MRT (h)	15.05	NA^a^
Half life (h)	10.43	NA^a^

*C*_max_: the maximum plasma concentration; *T*_max_: the time at which the *C*_max_ is achieved; AUC_last_: area under curve until the end of observation; AUC_∞_: area under curve from zero to infinity; MRT: mean residence time.

^a^ These values were unavailable because the plasma concentration of dexamethasone was still rising at the end of observation.

## Discussion

Metformin is a first-line drug for type 2 diabetes mellitus (formerly “non-insulin dependent diabetes mellitus”), and is one of the most commonly prescribed drugs worldwide. As a biguanide agent, metformin lowers both basal and postprandial plasma glucose (Scarpello & Howlett, 2008; Viollet et al., 2012). Metformin can be used as a monotherapy or in combination with other anti-diabetic agents, and in addition to lowering blood glucose levels, it may have additional health benefits including weight reduction, lowering plasma lipid levels, and prevention of some vascular complications (DeFronzo & Goodman, 1995).

Dexamethasone is one of the most commonly used systemic glucocorticoids and exhibits pleiotropic effects useful for treating a diverse range of disease including asthma, rheumatoid arthritis, systemic lupus erythematosus and acute kidney transplant rejection (Czock et al., 2005). Dexamethasone is also prescribed in order to lessen inflammation after dental treatment (Kim et al., 2009).

The plasma and biological half-lives of metformin hydrochloride are reported to be 6.2 and 17.6 h, respectively, but are also highly variable (Corti et al., 2007). The bioavailability of metformin is about 50–60%. On the other hand, the plasma and biological half-lives of dexamethasone are 100–300 min and 36–54 h, respectively (Montgomery et al., 1990) with an oral bioavailability of 70–78% (Loew et al., 1986). One of the great differences in the physicochemical characteristics of metformin and dexamethasone is their solubility in water. Specifically, metformin hydrochloride is highly soluble, permitting rapid dissolution from immediate-release tablets in the stomach; however, its passive distribution across cell membranes is limited by its low lipid solubility. In contrast, dexamethasone has a poor solubility in water and is highly lipophilic, resulting in difficult preparation of liquid formulations. Thus, several strategies have been utilized to deliver adequate amounts of dexamethasone, including complexation with cyclodextrin (Dilova et al., 2004).

Recently, dexamethasone delivery to ocular area has been studied extensively (Araki-Sasaki et al., 2016; Kalam 2016; Prieto et al., 2016). The routes of delivery were various including topical, oral and intravitreal injection. One of the purposes of these reports was the demonstration of sustained release of dexamethasone. The concentrations of dexamethasone were measured usually in the tear fluid, thus the direct comparison with the present study is impossible. However, the result of the present study could be considered quite promising from the perspectives of sustained release. On the other hand, metformin is usually administered by oral intake and the dose is much higher compared with 10 mg used in this study, although it has been compared with other diabetic drugs or with specialized vehicles (Cetin & Sahin 2016; Rhee et al., 2016). The metformin hydrochloride might not be a suitable candidate for the IMDDS used in the present study due to the requirement of high dose, but the results presented the need for slowing mechanism for this type of highly soluble drugs when administered via Osseogate.

Drugs administered through the Osseogate route have two possible pathways for diffusion, namely, through the vascular system in the bone marrow and lacunar–canalicular systems in bone. Distribution through the latter route is assumed to be negligible, although further individualized investigations are needed to determine the cortical bone permeability of specific drugs. The prototype IMDDS used in this study did not employ any active elements to expel the drug contents. Therefore, drug release was assumed to be due to simple diffusion at the interface between the drug cartridge and bone marrow, which was facilitated by the multiple holes in the IMDDS. In this way, the release patterns were considered to be largely dependent on the physicochemical characteristics of the loaded drug.

The results of the present study reflected this feature faithfully, with the release profiles of metformin hydrochloride and dexamethasone exhibiting completely distinct patterns that were highly consistent with the known pharmacokinetic properties of each drug. Specifically, compared to dexamethasone, the peak plasma concentration of metformin hydrochloride was reached early at the first sampling time point two hours after loading the drug, and may have actually been reached much earlier. Conversely, dexamethasone was detected through the end of observation period, whereas metformin hydrochloride was not detected in plasma after 72 h. Differences in drug solubility present a plausible explanation for this difference in plasma concentrations. In other words, highly soluble drugs like metformin hydrochloride might require additional modification to obtain a more sustained release profile and prolonged efficacy when using Osseogate. Nevertheless, both drugs in the present study met our criteria of a sustained release pattern using the prototype IMDDS.

In everyday practice, dentists frequently encounter patients with dental problems accompanied by one or more chronic systemic diseases (Umino & Nagao, 1993). Such systemic disease is usually identified when taking the medical history of patients, with the utmost consideration given to potential complications and contraindications of the disease during the treatment of dental problems. Recently, many kinds of systemic diseases have been found to be directly and indirectly associated with dental problems, especially periodontitis (Beck & Offenbacher, 2001; Kowall et al., 2015). However, the cause and effect relationships of these associations have not been completely elucidated (Kaur et al., 2009; Teeuw et al., 2010).

Among the existing array of drug delivery strategies, many are relevant to the oral and maxillofacial areas that are of primary interest in dentistry. Indeed, various types of scaffolds and bone morphogenic protein carriers to facilitate bone regeneration are being developed enthusiastically (Ramazanoglu et al., 2013). Dendrimers (Jia et al., 2014) and nanotubes (Losic et al., 2015) are also being investigated as materials for practical use in dentistry, as well as mucoadhesive patches for treating oral cancers (Holpuch et al., 2012). Of particular relevance to this study, implantable depots of certain drugs have also been introduced (Pehlivan et al., 2015). However, to the best of our knowledge, there have been no reports of an IMDDS utilizing the Osseogate route as a permanent gateway for painless drug delivery for general systemic disease control.

Titanium implants have been shown to have excellent biocompatibility and superb stability as far back as 50 years, and are currently the preferred choice for restorative options in dentistry (Park et al., 2011; Chung et al., 2013). Although there have been several attempts to expand the application of titanium implants out of the oral and maxillofacial area (Goiato et al., 2009), their most prevalent usage remains confined to dental restorations. Thus, the newly introduced IMDDS could open new horizons in the applications for titanium implants. Regarding longevity, IMDDSs are expected to have significant merit, since they do not experience any mechanical load if properly managed. In addition, installing an IMDDS in an oral environment has several advantages with regard to self-hygienic control and the ability to utilize well-established maintenance protocols generated from conventional dental implants (Gitto et al., 1994). However, there is a strong possibility that anatomic differences such as bone marrow distribution and intrabony circulation influence the drug absorption. Thus, further studies are recommendable to elucidate the different features according to the installation sites.

There are numerous challenges that must be overcome for practical use of the Osseogate route. Foremost, the release profile of the prototype IMDDS described in this study relied entirely upon the physicochemical properties of the specific drug being delivered. Thus, further development of mechanisms for controlled release of target drugs is recommended to achieve better delivery profiles. In addition, dosing requirements for specific drugs is another issue of the Osseogate route, because the available space in the IMDDS is somewhat limited. Nevertheless, the attractive features of this novel route should not be underestimated, and warrant further investigation for delivery of various drugs for the treatment of disease.

## Conclusions

Through the novel drug delivery route, Osseogate, comparison of release profiles was performed between water soluble metformin hydrochloride and minimally soluble dexamethasone using IMDDS. Although the sustained release was detected in both groups, metformin showed more rapid peak concentration and earlier end than dexamethasone. The results of the present study suggested, without additional controlling mechanism, drug release profile through IMDDS be largely dependent on the drug’s physicochemical properties, especially the solubility.
